# High Temperature Performance of Concrete Confinement by MWCNT Modified Epoxy Based Fiber Reinforced Composites

**DOI:** 10.3390/ma15249051

**Published:** 2022-12-18

**Authors:** Lakshmi Joseph, Mini K. Madhavan, Karingamanna Jayanarayanan, Alessandro Pegoretti

**Affiliations:** 1 Department of Civil Engineering, Amrita School of Engineering, Amrita Vishwa Vidyapeetham, Coimbatore 641112, India; 2 Department of Chemical Engineering and Materials Science, Amrita School of Engineering, Amrita Vishwa Vidyapeetham, Coimbatore 641112, India; 3 Center of Excellence in Advanced Materials and Green Technologies (CoE-AMGT), Amrita School of Engineering, Amrita Vishwa Vidyapeetham, Coimbatore 641112, India; 4 Department of Industrial Engineering, University of Trento, Via Sommarive 9, 38123 Trento, Italy

**Keywords:** concrete columns, fiber reinforced polymers, MWCNT, hybrid fiber wrapping, compressive strength, thermal durability

## Abstract

The conventional method of fiber reinforced polymer (FRP) wrapping around concrete columns uses epoxy as the binder along with synthetic or natural fibers such as carbon, glass, basalt, jute, sisal etc. as the reinforcement. However, the thermal stability of epoxy is a major issue in application areas prone to fire exposure. The current work addressed this major drawback of epoxy by modifying it with a nanofiller, such as multiwalled carbon nanotubes (MWCNT), and reinforcing it using basalt and sisal fibers. The effect of exposure to elevated temperature on the behavior of concrete cylinders externally confined with these FRP systems was analyzed. Three types of specimens were considered: unconfined; confined with sisal fiber reinforced polymer (SFRP); and confined with hybrid sisal basalt fiber reinforced polymer (HSBFRP) specimens. The test samples were exposed to elevated temperature regimes of 100 °C, 200 °C, 300 °C and 400 °C for a period of 2 h. The compressive strengths of unconfined specimens were compared with various confined specimens, and from the test results, it was evident that the mechanical and thermal durability of the FRP systems was substantially enhanced by MWCNT incorporation. The reduction in the compressive strength of the FRP-confined specimens varied depending on the type of the confinement. After two hours of exposure at 400 °C, the compressive strength corresponding to the epoxy–HSBFRP-confined specimens were improved by 15%, whereas a 50% increase in strength corresponding to MWCNT-incorporated epoxy–HSBFRP-confined specimens was observed with respect to unconfined unexposed specimens. The MWCNT-modified epoxy-incorporated FRP-confined systems demonstrated superior performance even at elevated temperatures in comparison to unconfined specimens at ambient temperatures.

## 1. Introduction

Reinforced polymer (FRP) composites are identified as the most promising material in the area of structural strengthening and retrofitting. FRPs are mainly used as an external confinement in structures particularly in columns and beams [1]. The ease of installation, corrosion resistance, high strength to weight ratio and improved tensile strength are the most appealing attributes of FRPs, which further qualifies these as materials of immense relevance in structural rehabilitation [2]. Using an epoxy resin adhesive, the fiber systems are attached to the external surfaces of structures in the form of an additional confining reinforcement [3]. Epoxy resin is a type of thermosetting polymer adopted as a matrix in FRP composites. The high strength-to-weight ratio, stiffness and excellent chemical properties qualify it as a suitable choice as an FRP matrix [4]. During its lifespan, FRP composites are often vulnerable to different harsh environments, and their failure modes vary depending on these conditions [5]. The addition of suitable fillers enhances both the mechanical and durability properties of epoxies [6]. The incorporation of nanomaterials, particularly multi-walled carbon nanotubes (MWCNT) is known to enhance the various mechanical and thermal aging properties of epoxy. An effective surface for filler–matrix interaction and stress transfer are contributed by the large surface area of MWCNT [7]. The nano modifications are capable of reducing the surface wettability and water uptake of epoxy nanocomposite and thereby significantly improving the durability of the composite [8]. However, at higher concentrations of MWCNT, van der Waals forces of attraction cause clustering of MWCNT. Hence the degree of dispersion of MWCNT in epoxy affects the fracture behavior and mechanical properties of composites [9,10].

Carbon, glass and aramid are the most commonly used fibers for structural retrofitting and strengthening [11,12,13]. The usage of natural fiber in FRP as a replacement for synthetic fibers is a major research area that has emerged in recent times. The usage of eco-friendly natural fibers can be viewed as a sustainable and renewable solution [14]. In India, an extensive variety of natural fibers such as jute, sisal, coir etc., are abundantly available. Their medium tensile and flexural properties, along with their greater impact properties, make them a suitable choice in FRP systems [15,16]. However, low durability of natural fibers is a major disadvantage when compared to synthetic fibers [17,18,19]. The hybridization of natural fibers with synthetic fibers is a viable solution to this problem. There is a combined effect of the positive elements of different fibers on hybridization. The composites made up of the hybridization of sisal, jute and abaca fibers with GFRP and CFRP showed superior properties when compared with natural fiber systems [20,21]. Paul et al. [22] investigated the axial and ductile behavior of jute FRP and polyester FRP-confined specimens. The FRP confinement could considerably improve the strength and ductile behavior of columns since both the fibers have better strength and non-corrosive nature. Padanattil et al. [23] assessed the behavior of hybrid FRP systems containing sisal and glass fibers. It was found that the hybridization of natural and synthetic fibers yielded superior properties and even had the potential to replace conventional CFRP systems in structural strengthening and retrofitting. Huang et al. [24] studied the performance of polyester fiber sheets as FRP confinement. It was found that polyester–FRP enhanced the ductile behavior of confined specimens, and improved their compressive strength. When polyester FRP was hybridized with jute FRP, there was a synergy of the positive features of both the fibers. The hybrid FRP system showed superior mechanical and durability characteristics. An experimental study by Joseph et al. [25] concluded that superior properties were exhibited by MWCNT-incorporated epoxy-based sisal–basalt FRP confinement and that the low strength exhibited by the natural FRP system when compared with an artificial FRP system could be enhanced by epoxy modifications.

When exposed to fire, FRPs are prone to burning of the matrix, which is commonly epoxy, and the eventual damage of the confinement. Therefore, the usage of FRP in structural confinement has been questioned due to their vulnerability at elevated temperatures. Limited studies were carried out on the performance of FRP-confined systems in elevated temperatures. Li and Xian [26] studied the influence of elevated temperature on the thermal and microstructure properties of CFRP plates prepared with bisphenol-A epoxy matrix and hydantoin epoxy matrix. The study revealed that the allowable exposure temperature with desirable tensile stiffness was 200 °C. The mechanical properties of the nanocomposites are of immense significance in engineering applications. Several studies [27,28,29,30] were carried out to tailor the properties of fiber-reinforced and nano composites based on the type and weight fraction of the fillers. Hollaway [31] opined that temperature is a major parameter that influences the behavior of FRP materials. High temperature changes the arrangement of epoxy polymer chains and subsequently causes its degradation. The performance of FRP when exposed to elevated temperatures is influenced mainly by the glass transition temperature (T_g_) of epoxy. When exposed to an elevated temperature above the T_g_ value, there is a considerable reduction in the performance of FRP. The behavior of FRP-confined specimens when exposed to high temperatures regimes was investigated by Al-Salloum et al. [32]. CFRP and GFRP-confined cylinders were exposed to 100 °C and 200 °C temperature regimes and a considerable reduction in strength was observed, especially at 200 °C. Yaqub et al. [33,34] analyzed the performance of reinforced square columns confined with single layers of GFRP and CFRP post heat exposure. Upon exposure to 500 °C, the ultimate strength of unconfined specimens reduced considerably while the confined specimens exhibited some resistance due to the confining effect shown by the FRP layers, even after dilation. The increment in the FRP layers had a considerable influence on the performance of the confining systems.

Sherif et al. [35] studied the performance of CFRP and GFRP-confined specimens when exposed to temperatures of 100 °C, 200 °C, and 300 °C for a period of 1, 2, and 3 h. The unconfined specimens did not have much of an impact when exposed to high temperatures while there was a considerable reduction in the confinement effect provided by FRP-confined specimens upon exposure to higher temperature. Trapko [36] investigated the behavior of CFRP and fiber reinforced cementitious matrix (FRCM) confined specimens when exposed to 40 °C, 60 °C, and 80 °C for a period of 24 h. The CFRP-confined specimens failed due to the sudden rupture of FRP layers while FRCM-confined specimens failed by the debonding of FRCM meshes. The service temperature had a noteworthy impact on the failure of CFRP-confined specimens due to the softening of polymer at elevated temperatures for a longer period. The bond developed between the FRP layers and core surface deteriorate quickly when exposed to temperatures greater than T_g_ and could ultimately lead to the FRP debonding from the concrete core.

It is evident from the literature survey that an in-depth thermal durability study of sisal fiber-reinforced polymer (SFRP) and hybrid sisal basalt fiber-reinforced polymer (HSBFRP) as an external confinement in structural elements has not been explored in detail. The present study particularly aimed to investigate the axial compressive behavior of epoxy-based SFRP and HSBFRP-confined systems filled with MWCNT once exposed to different elevated temperature regimes. In the investigation, the unconfined and confined concrete specimens (with FRP confinements) were exposed to elevated temperature regimes of 100 °C, 200 °C, 300 °C, and 400 °C for a period of 2 h, after which they were tested for axial compression until failure. The maximum temperature was selected as 400 °C as it is reported that the epoxy loses its strength above 200 °C, and that for concrete it is in the vicinity of 400 °C. Furthermore, the ultimate load carrying capacity, failure modes, stress–strain response, influence of MWCNT incorporation towards thermal resistance, and ductile behavior of the confined specimens were compared with the unconfined specimens.

## 2. Experimental Program

### 2.1. Overview of Test Program

**Figure 1:**
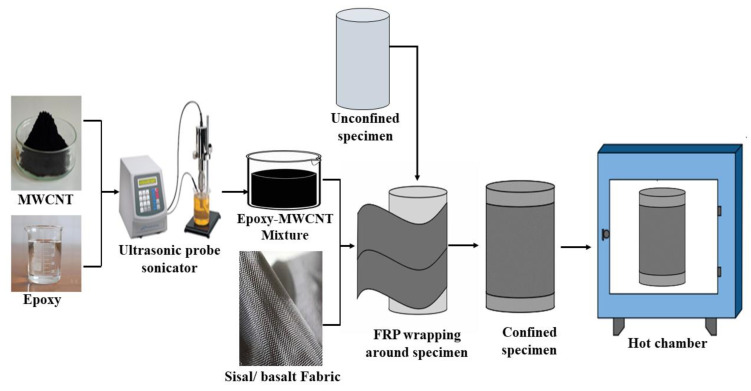
Schematic representation of test program.

A total of 75 plain concrete cylinders were investigated under axial compressive loading to analyze the effect of FRP confinement after exposure to different temperature regimes, out of which 15 cylinders were unconfined control specimens and 60 cylinders were confined using sisal and sisal basalt hybrid FRP systems. The circular column specimens were 300 mm in height and 150 mm in diameter. After 28 days of curing the cylinders were dried under room environmental conditions and the radial surfaces were cleaned to remove dust and impurities. A suitable adhesion between the inner concrete core and outer FRP sheets was attained using high-performance epoxy filled with MWCNT-incorporated resin. A thin coating of epoxy matrix was initially applied over the concrete surface and the fiber layers were completely impregnated with epoxy. Subsequently the FRP layers were wrapped around the concrete cylinders by hand layup. In case of hybrid FRP, sisal FRP layers were wrapped as the inner layers followed by basalt FRP layers as outer layers [17]. Debonding or slippage between the FRP layers was prevented by providing a 150 mm overlap length for every layer. After which the concrete cylinders were exposed to various temperature regimes as 100 °C, 200 °C, 300 °C, and 400 °C separately for a period of 2 h. The schematic representation of the test program is illustrated in Figure 1.

### 2.2. Material Properties

#### 2.2.1. Concrete

The concrete cores were cast with OPC 53 Grade cement, fine aggregate (FA) and coarse aggregates (CA) with a specific gravity of 3.17, 2.7 and 2.67 respectively. As per IS 10262-2009 the concrete cylinders were designed and casted. A mix proportion of 1:1.5:2.58 by weight of cement, FA, and CA along with a water/cement ratio of 0.45 as explained in Table 1 was maintained throughout the study.

#### 2.2.2. FRP Sheets, MWCNT and Resin

A high-performance two-part epoxy resin was the polymer matrix. It consists of resin (LY 556) and hardener (HY 991) mixed in a ratio of 100:15 (Part A: Part B) by weight as suggested by the manufacturer. MWCNTs modified with carboxylic acid (–COOH) having 97% purity, 2–10 microns average length, and specific surface area 250–260 m^2^/g from Platonic Nanotech Private Ltd., Mahagama, India, were used. MWCNTs were evenly dispersed in epoxy resin for 30 min using a 20 kHz frequency ultrasonic probe sonicator.

Bi-directional sisal fabric of 300 g/m^2^ was used as the inner layer of the external confinement. The thickness of sisal fabric ranged between from 0.8 to 1 mm with a density of 1580 kg/m^3^. The easy availability, economical, and medium stiffness of sisal fiber qualify it as a promising choice. Sisal fibers were subjected to alkaline treatment by immersion in NaOH solution and further drying to room temperature for 72 h. The treatment was capable of improving the surface roughness of the fiber and further resulting in enhanced mechanical interlocking. Commercially available basalt fiber sheets of 380 g/m^2^ with a density of 2630 kg/m^3^ as provided by the manufacturer were used as an outer layer of confining systems. Both the sisal and basalt fibers in the form of continuous sheets were purchased from Go green Products, Chennai, Tamil Nadu. Apart from the strengthening aspect, the outer layer of basalt provides a protection to the inner natural sisal fiber confinement against the atmospheric effects.

### 2.3. Temperature Regimes

The following samples were tested: 15 unconfined cylinders, 15 cylinders wrapped with two layers of epoxy-based SFRP sheets, 15 cylinders wrapped with two layers of epoxy-based SFRP sheets filled with MWCNT, 15 cylinders wrapped with four layers of epoxy-based hybrid SBFRP sheets, 15 cylinders wrapped with four layers of epoxy-based hybrid SBFRP sheets filled with MWCNT. Five temperatures were considered during the study to analyze the influence of elevated temperature on the confinement effect. The first regime denotes the room temperature and after which all the specimens were exposed to 100 °C, 200 °C, 300 °C, and 400 °C separately for a period of 2 h. After the high temperature exposure, the cylinders were tested under uniaxial compressive mode. These temperature regimes and the corresponding specimen nomenclature details are summarized in Table 2. An alphanumeric specimen nomenclature was adopted. For example, C-E1C2S2B200: the initial letter C means concrete, the second letter E denotes the presence of epoxy, 1C indicate the weight percentage of MWCNT (1% in this case), 2S refer to the number of sisal FRP layers, 2B indicate the number of basalt FRP layers (2B) and 200 denotes the exposure temperature.

### 2.4. Test Procedure

The entire study was divided into two phases. In the first phase, the various properties of epoxy were analyzed, whereas in the second phase, the effect of FRP confinement on concrete was studied.

#### 2.4.1. Material Testing

The tensile strength of the epoxy and multiscale composites were analysed as per ASTM D3039. The dynamic mechanical analysis was conducted in the temperature range between 27 °C to 240 °C at a heating rate of 5 °C/min in a Perkin Elmer DMA8000, maintaining a displacement range of +/− 1 to 1400 µm. The damping factor (tan δ) was recorded by a three-point bending method at a frequency of 1 Hz. In a temperature range of 0 °C to 600 °C along with a nitrogen atmosphere by thermogravimetric analysis, the thermal degradation behavior of the epoxy and multiscale composites were analyzed.

#### 2.4.2. Structural Testing

The confined and unconfined columns were tested for compressive strength under a monotonical axial compression load in a UTM until failure. To ensure uniform loading over the cylinders, steel plates of 5 mm thickness were placed above and below the test specimens while loading. The specimens were tested at a constant displacement rate of 0.2 mm/min. Since a high axial displacement is expected, Linear variable differential transducers (LVDTs) having a capacity of 50 mm were used to record axial strains on the confined and unconfined concrete specimens. For each sample, at least three specimens were tested.

## 3. Experimental Results and Discussion

### 3.1. Phase 1: Tests on MWCNT Incorporated Epoxy Based FRP Composite

#### 3.1.1. Mechanical Properties

**Figure 2:**
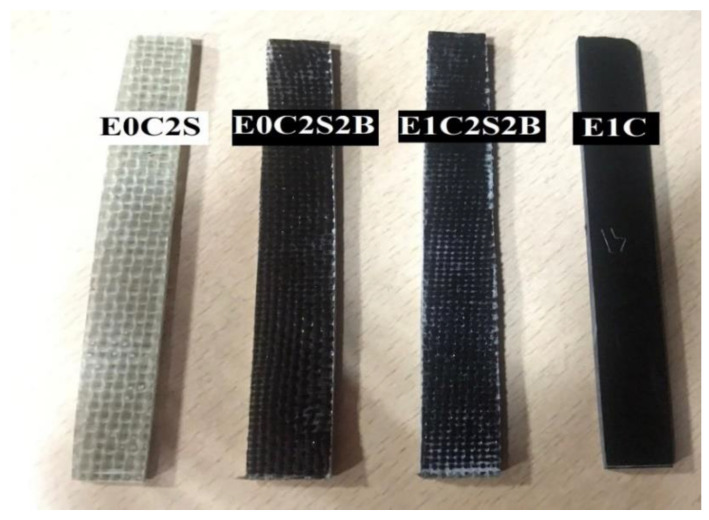
Epoxy and epoxy multiscale composites.

The tensile strength exhibited by epoxy and multiscale composites were determined as per ASTM D3039 by preparing different test coupons. The flat coupons 100 mm in length, 10 mm in width and 3 mm in thickness were tested (Figure 2). The specimens were prepared as neat epoxy, epoxy filled with 1 wt.% of MWCNT, epoxy with different fiber layers and epoxy multiscale composites with different fiber layers and filled with 1 wt.% of MWCNT. The epoxy and multiscale composites were designated as ExCySzB where x denotes the weight percentage of MWCNT in epoxy resin and y and z denotes number of sisal and basalt fiber sheets respectively in the epoxy resin. The samples were prepared by hand layup method followed by room temperature curing for seven days prior to testing using an INSTRON 502 UTM at a crosshead speed of 1 mm/min. From the test results (Table 3), it is inferred that there is a noteworthy improvement in tensile strength and modulus with the MWCNT incorporation. The neat epoxy samples showed 40 MPa tensile strength and upon MWCNT addition there was an improvement of tensile strength by 50% and 170%, respectively, corresponding to 1 wt.% of MWCNT-incorporated epoxy samples (E1C) and 1 wt.% of MWCNT-incorporated epoxy multiscale samples (E1C2S2B). This enhancement in the tensile strength and modulus may be credited to the superior load bearing characteristics of MWCNT-incorporated epoxy samples. Considering the nanosized dimensions of MWCNT, the specific surface area is high for the interaction with the epoxy matrix. The uniform dispersion of MWCNT together with the large interfacial area within the epoxy and MWCNT aids in the improvement of mechanical properties of nanocomposites.

**Figure 3:**
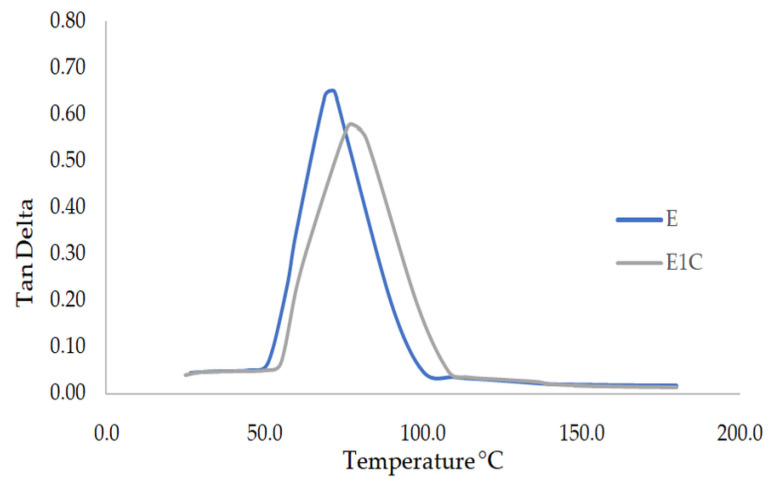
tan delta curve for epoxy nano and multiscale composites.

The viscoelastic properties of the composites were assessed by dynamic mechanical analysis (DMA). The tan delta parameter obtained from DMA is often analyzed to understand the glass transition temperature of polymers. Tan delta may be defined as the viscous to elastic response ratio of viscoelastic materials. Tan delta values corresponding to epoxy (E) and epoxy-MWCNT nanocomposites (E1C) are shown in Figure 3. The peak tan delta values corresponding to nano composites are reduced by the addition of MWCNT when compared with neat epoxy. Upon filler addition, the tan delta peak values usually decrease as already reported in literature [37]. From the results it is evident that there is a shift in the tan delta peak to higher temperatures which is an indication of the improvement in the T_g_. This is because of the restrictions offered by MWCNT for the slippage of epoxy polymer chains. Glass transition temperature is a measure of the transition of a polymer from a glassy to a rubbery state. The presence of MWCNT hinders the movement of epoxy polymer molecules at elevated temperature. Furthermore, with the addition of nano filler, the area under the tan delta curve increases as a sign of the damping characteristics of the MWCNT-incorporated epoxy system. The reason may be the higher degree of molecular arrangement in nano-incorporated composites, which may be due to the better dispersion of MWCNT within the epoxy matrix [9]. The broadening of the peak for E1C reveals the superior relaxation properties of the composites. The positive shift in the glass transition temperature (T_g_) of E1C with respect to epoxy samples indicates an immobilizing effect played by MWCNT on the epoxy resin molecules.

#### 3.1.2. Thermogravimetric Analysis

**Figure 4:**
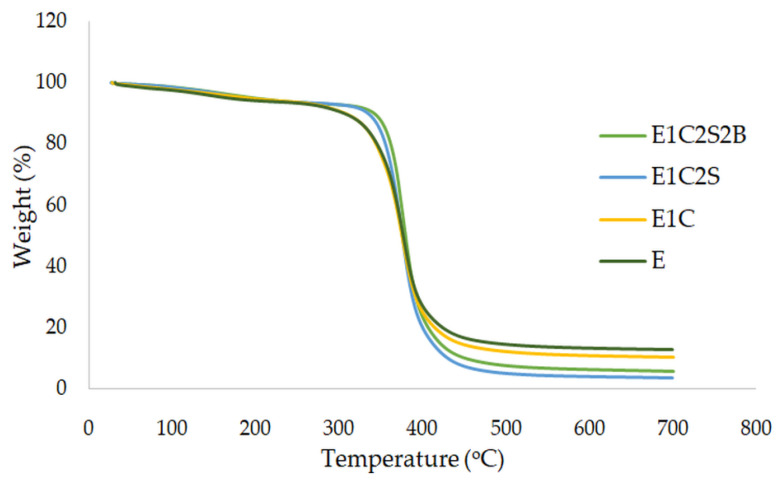
Dynamic TG profiles of composites.

**Figure 5:**
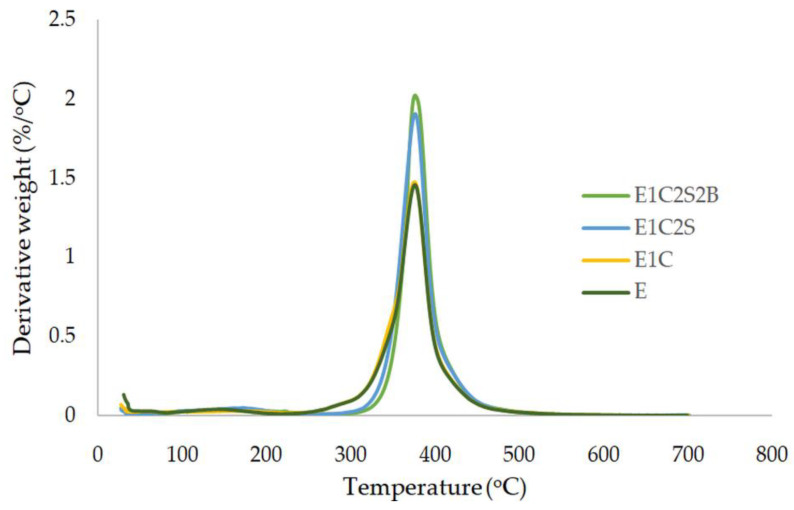
Derivative thermogram of composites.

Thermogravimetric analysis (TGA) was carried out to analyze the thermal decomposition characteristics of epoxy and epoxy multiscale composites. The variation of composite modifications with temperature of E, E1C, E1C2S and E1C2S2B samples is presented in Figure 4. Likewise, the derivative thermograms of these curves are illustrated in Figure 5. The temperature at which the bulk decomposition of the matrix commences is at the shoulder of these curves. The decomposition temperature of the epoxy is improved due to the inclusion of MWCNT, since it restricts the evolution of volatile matter from the polymer contributing to its degradation. As a result of the addition of MWCNT, an insulating layer is created for the transfer of volatile matter, which in turn raises the degradation temperature. When compared with neat epoxy specimens, E1C2S2B nanocomposite displayed a shift of the shoulder to higher temperature. The investigation demonstrated that MWCNT incorporation to epoxy-based hybrid sisal–basalt epoxy composite elevates the onset temperature of degradation. The uniform dispersion of MWCNT could delay the T_onset_ and thereby enhance the thermal stability [38]. In the case of E1C2S2B, the decomposition starts at 382 °C and temperature at which the decomposition rate is maximum is observed at 420 °C. While neat epoxy composites start decomposing at 342 °C and reach their maximum decomposition at 408 °C. The decomposition of polymer chains near the MWCNT is gradual, and it shifts dT_max_ to elevated temperatures. The uniform heat dissipation within the matrix is ensured by nanotubes due to their thermal conductive nature, which further improves the thermal stability of composite. The durable interface developed between epoxy and MWCNT can delay the disintegration of matrix at higher temperatures. When the heat diffusion progresses, the temperature at which the maximum weight loss occurs may move to higher temperatures which in turn is due to the thermal barrier created by the addition of nano causing a delay in the volatilization. It could be concluded that TGA studies specified that the combined effect created due to MWCNT and hybrid fiber systems promotes its application in elevated temperature environments.

### 3.2. Phase 2: Failure Modes and Compressive Behavior of Concrete Specimens

#### 3.2.1. Unconfined Specimens

**Figure 6:**
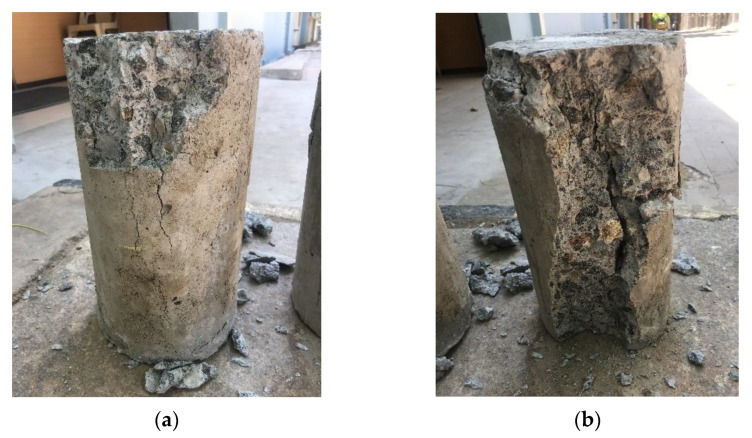
Failure pattern of unconfined specimens (**a**) CS (**b**) CS 400.

The unconfined cylinders were exposed to five temperature regimes viz. room temperature, 100 °C, 200 °C, 300 °C, and 400 °C. The failure of unconfined specimens under axial loading after exposure to high temperatures was predominantly due to the splitting of outer concrete layers. Figure 6 displays the failure patterns of unconfined cylinders after compression testing. Cracking along with concrete spalling was observed over the concrete surfaces [39]. The average unconfined concrete strength at ambient temperature was 14.9 MPa as seen in Table 4. The core strength was reduced by 5%, 13%, 18% and 30% at 100 °C, 200 °C, 300 °C, and 400 °C respectively. It may be noticed that exposure to elevated temperatures had a considerable impact on the strength and performance of the unconfined specimens. Also, at specific elevated temperatures (i.e., 400 °C), there is a steady loss in concrete strength. It is reported that when the temperature reaches to 300 °C the evaporation of the chemically bound water occurs which results in a decrease in the compressive strength of concrete. As temperature is further increased to 400 °C, decomposition of portlandite occurs. This resulted in noticeable strength loss at elevated temperatures. Also, it could be inferred that in the eventuality of a temperature rise due to fire or any emergency, the concrete tends to lose its strength with respect to time.

#### 3.2.2. SFRP Confined Specimens

**Figure 7:**
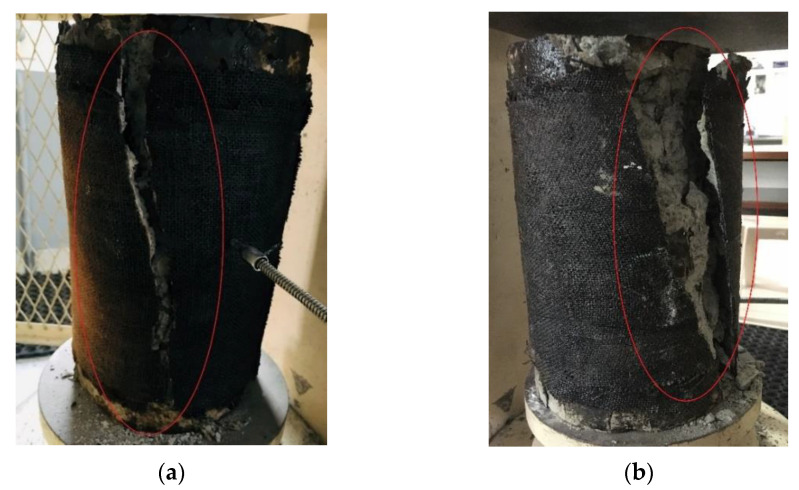
Failure patterns of confined SFRP specimens exposed to elevated temperature: (**a**) C-E0C2S400; and (**b**) C-E1C2S400.

This section demonstrates the behavior of specimens confined with two layers of epoxy SFRP sheets and MWCNT-incorporated epoxy-modified SFRP sheets when exposed to different temperature regimes for 2 h. In Figure 7, it is evident that in confined specimens the epoxy–FRP wrapping is severely damaged when exposed to high temperature. The FRP jackets of the exposed specimens when tested under axial compression were ruptured. The failure pattern of the epoxy-confined specimens exposed to elevated temperature was characteristically accompanied with an exploding sound and was abrupt when compared to unexposed cylinders, due to the disintegration of epoxy at elevated temperatures. On the contrary, when MWCNT-incorporated SFRP specimens were considered, the complete disintegration of epoxy over the layers was not noticed. All the exposed SFRP-wrapped specimens failed by FRP rupture irrespective of exposure time or temperature rise. The epoxy-based SFRP-confined specimens started to deteriorate after 100 °C and the MWCNT-incorporated epoxy modified SFRP wrapped cylinders did not exhibit any severe deterioration when exposed to 100 °C temperature rise. However, the neat epoxy-confined cylinders exposed to a heating regime of 200 °C and above showcased some worsening of the FRP layers due to epoxy melting and charring. The MWCNT-incorporated SFRP-confined specimens improved the performances under axial load. Cracking sounds were overheard before failure as an indication of stress transfer between the concrete core and FRP wraps, and the failure pattern shows the full adherence of the wrapping with concrete (Figure 7b). A gradual failure characterized by concrete crushing tailed by the SFRP jacket rupture, at the middle part of the specimen could be observed.

The results of compression test of SFRP-confined specimens are listed in Table 5. It can be observed that the SFRP confinement of cylinders enhanced the compressive strength of concrete core. This increment ranged between 18% and 9% corresponding to room temperature and elevated temperature for epoxy-based SFRP-confined specimens, while the strength increased between 59% to 23% corresponding to room temperature and elevated temperature for MWCNT-incorporated epoxy-SFRP-confined specimens. The enhanced strength may be credited to the MWCNT modification of epoxy. Compression test results indicated that temperature level had a significant influence on the confinement effect of SFRP wraps.

**Figure 8:**
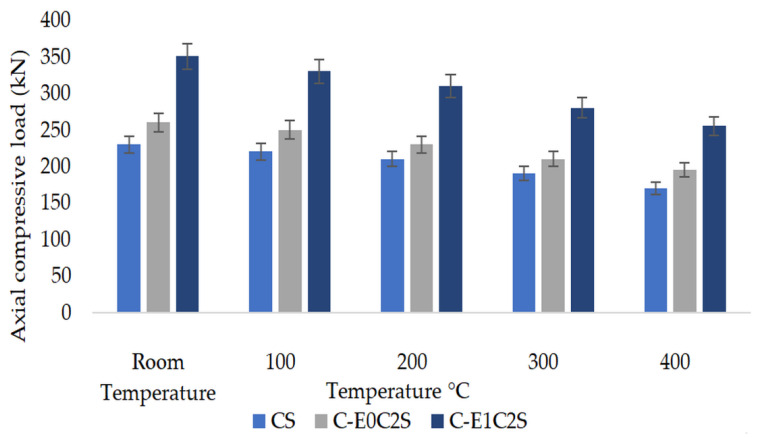
Maximum compressive load of unconfined and SFRP specimens exposed to elevated temperatures.

The confined specimens exposed to high temperature regimes exhibited lower strength properties corresponding to the same specimens tested under room temperature conditions (Figure 8). The rate of strength loss is higher beyond 200 °C as evident from the results.

#### 3.2.3. HSBFRP Confined Specimens

**Figure 9:**
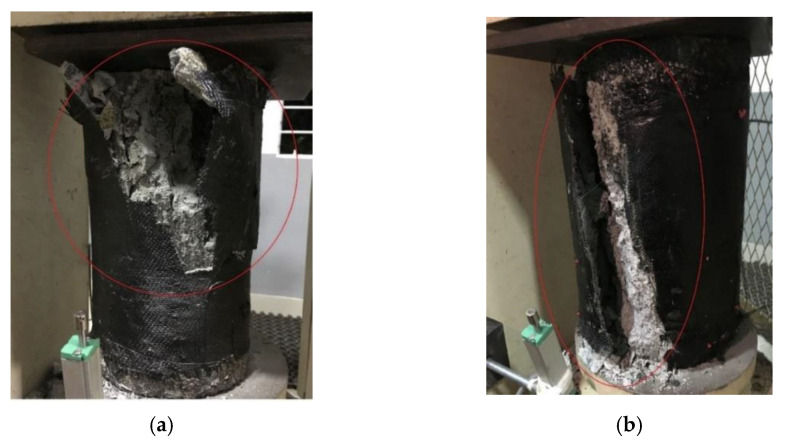
Failure pattern of confined HSBFRP specimen exposed to elevated temperature: (**a**) C-E0C2S2B400; and (**b**) C-E1C2S2B400.

The behavior of concrete cylinders wrapped with two layers of epoxy HSBFRP sheets and epoxy-modified HSBFRP filled with MWCNT sheets when exposed to different temperature regimes for 2 h is reported here. The failure modes exhibited by HSBFRP specimens were almost similar to that of the SFRP-confined specimens. The failure mechanisms exhibited by confined specimens were sudden and explosive as an indication of the rupture of HSBFRP wraps. The MWCNT incorporation in epoxy provided a greater resistance to the hybrid FRP layers under elevated temperature. Even when exposed to elevated temperature the hybrid FRP layers are found to be adhering to the concrete surface maintaining the confinement effect. The failure pattern exhibited by HSBFRP-confined specimens followed an explosive nature along with a high energy dissipation irrespective of the exposure temperature [40]. Figure 9 shows the failure pattern of epoxy-based and MWCNT-incorporated HSBFRP wrapped specimens after failure.

**Figure 10:**
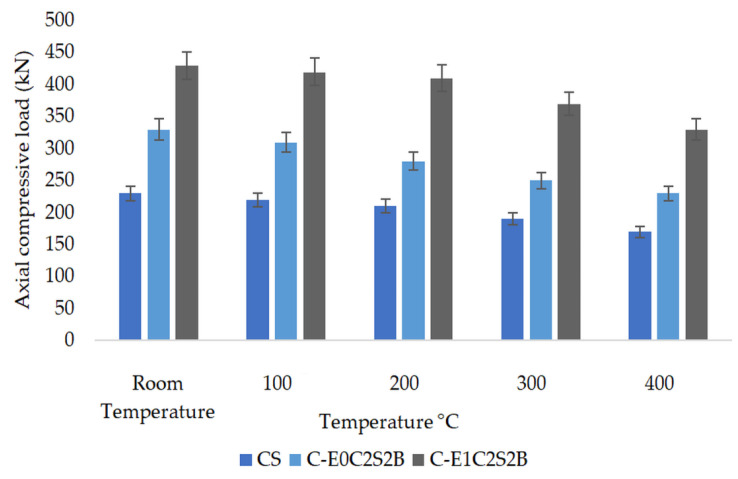
Maximum compressive load of unconfined and HSBFRP specimens exposed to elevated temperatures.

The loss in compressive strength for HSBFRP-confined cylinders exposed to various temperature conditions when compared with unconfined specimens is illustrated in Figure 10. HSBFRP confinement could considerably enhance the compressive strength of concrete core. From Table 6 it could be noted that at a given time, with increase in temperature the confined concrete strength decreases. The trend of compressive strength when exposed to elevated temperature is not quite similar to that of SFRP-confined specimens. The strength ranged between 50% to 14% corresponding to room temperature and elevated temperature for epoxy–HSBFRP-confined specimens, while the strength ranged between 95% to 50% corresponding to room temperature and elevated temperature for MWCNT-incorporated epoxy–HSBFRP-confined specimens. Regardless of the temperature condition, all the MWCNT-incorporated epoxy wrapped cylinders exhibited superior compressive strengths when compared with the epoxy confined specimens. This leads to the conclusion that, in case of any accidental fire, or such conditions, the concrete will lose its strength quickly. But the concrete confined with epoxy-based FRP and MWCNT-modified epoxy-based FRP provides a better protection to these elevated temperatures. The study inferred that superior thermal protection is observed for MWCNT-based HSBFRP specimens where the loss of strength is not appreciable due to the enhanced thermal degradation resistance as presented in Section 3.1.2.

### 3.3. Comparison of Axial Compressive Results

**Figure 11:**
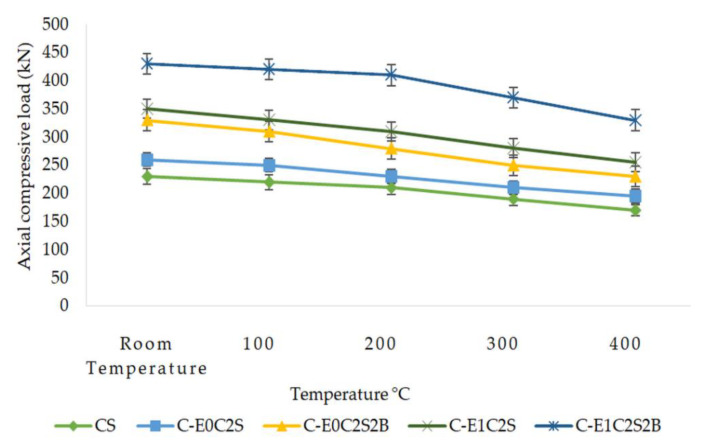
Maximum compressive load comparison of unconfined and confined specimens exposed to elevated temperatures.

Figure 11 displays the compressive strength variation corresponding to unconfined, SFRP-confined, and HSBFRP-confined specimens when subjected to different elevated temperature conditions. It could be observed that, with a rise in temperature the compressive strength reduces for all the specimens. Furthermore, the MWCNT-incorporated epoxy-HSBFRP-confined specimens exhibited extreme compressive strength, after that the epoxy-based HSBFRP-confined specimens and SFRP-confined specimens in that order. The increase in temperature had a remarkable influence on the strength properties particularly for the SFRP-confined specimens. However, MWCNT-incorporated epoxy-HSBFRP-confined specimens performed better in all temperature regimes. When unconfined specimens are considered, the strength reduction was particularly due to the concrete spalling, while the confined cylinders are considered, the strength degradation developed by reason of the progressive damage of the bond within concrete and different layers of FRP. The decrease in strength was predominant when confined specimens were exposed to 300 °C and above. Also, from the test results it was evident that epoxy-based confinements exhibited more severe damage than the MWCNT-modified epoxy-based confinements when they were exposed to elevated temperature.

### 3.4. Confining Effectiveness

The unconfined concrete strength is denoted as f_co_ while the confined concrete strength is denoted as f_cc_. The confinement effectiveness ratio corresponding to confined concrete is taken as f_cc_/f_co_ [23]. When the responses of confined and unconfined specimens are compared, it may be noticed that both SFRP and HSBFRP confinement is active in enhancing the axial load carrying capacity of concrete core. The MWCNT modification of epoxy further improved the confinement effectiveness. The effectiveness in terms of strength (f_cc_/f_co_) was approximately 1.96 for a MWCNT-incorporated epoxy HSBFRP jacketing at room temperature. Confinement effectiveness showed a decreasing trend with rise in temperature. Epoxy–HSBFRP exhibited a confining effectiveness of 1.35 at 400 °C while the MWCNT-incorporated epoxy HSBFRP confinement had its minimum effectiveness of 1.91 at 400 °C. In the case of MWCNT-incorporated epoxy SFRP, the maximum and minimum confining effectiveness was found to be 1.45 and 1.18 at room temperature and 400 °C respectively, as clear evidence of the enhanced confinement effectiveness of concrete on MWCNT-incorporated FRP confinement. The confining effectiveness is also influenced by the count of FRP layers used for wrapping. For a four-layer hybrid system, the confining effectiveness exhibited by HSBFRP specimens was considerably greater than the SFRP specimens at ambient and elevated temperatures.

### 3.5. Stress-Strain Curves and Ductility Behavior

**Figure 12:**
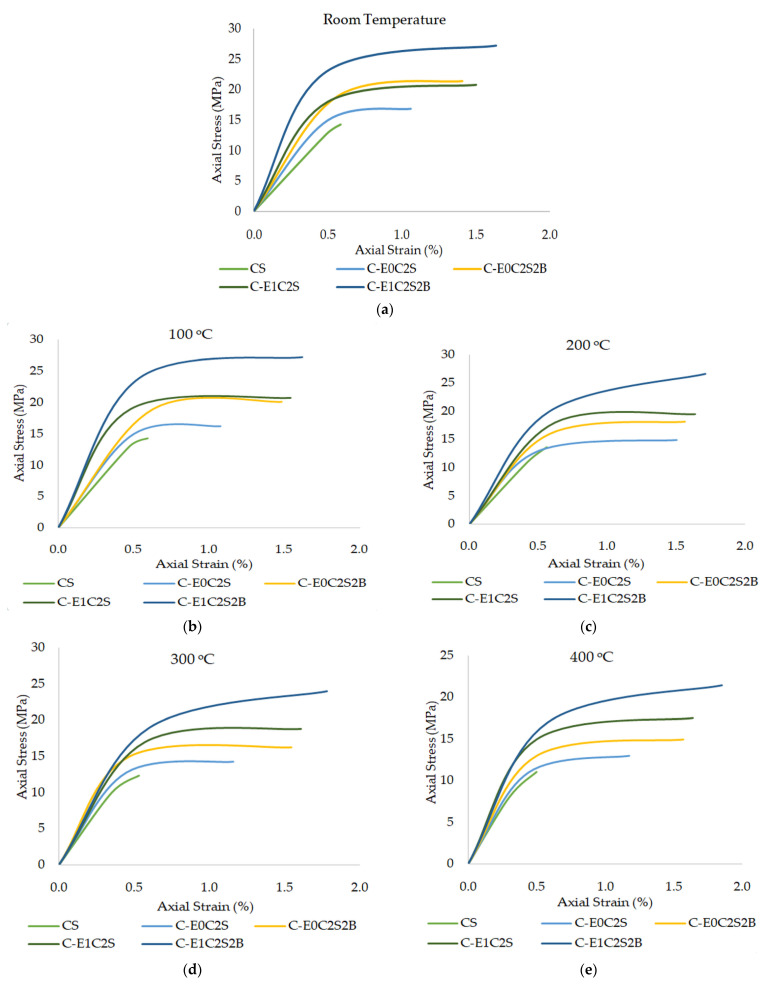
Axial stress–strain curves of cylinders exposed to different temperature regimes: (**a**) room temperature; (**b**)100 °C; (**c**) 200 °C; (**d**) 300 °C; and (**e**) 400 °C.

The representative stress–strain curves for various FRP-jacketed and unconfined specimens are shown in Figure 12. The stress–strain curves for confined specimens exhibited different stages. The initial stage depicts a linear tendency similar to the unconfined concrete. In the next phase a changeover was observed wherein the stresses in the concrete core exceeded the limit and a confining pressure was transferred to the FRP layers. In the final stage, a linear drift with decreased slope is observed. The confinement effect offered by FRP is mainly activated in the last two stages of the stress–strain curve [41]. When unconfined specimens are considered, the failure occurs at the termination of initial stage due to the brittle behavior of concrete while FRP-confined specimens exhibit a ductile failure mode. Along with the HSBFRP confinement, the SFRP confinement has also showcased a considerable enhancement in ductility. The strain results of the specimens exposed to different temperature conditions including ambient condition, 100 °C, 200 °C, 300 °C, and 400 °C, and the numerical test summary are presented in Table 7.

The stress–strain curves displayed a consistent behavior, however, there was an increment in strain percentages with temperature rise from 100 °C to 400 °C. The stress and strain increased in a linear manner initially and once ultimate stress was attained, there was sharp increment in strain even for a small increase in stress. This may be credited to the FRP softening which in turn resulted in the decrease in the confinement effect of concrete when exposed to elevated temperature.

Ductility is a significant property that permits a structure to undergo large deformations prior to ultimate failure [14]. Due to the high energy dissipative nature of ductile materials, proper warnings are obtained before catastrophic failure. It is a significant feature especially during the design and deployment of earthquake-resistant structures. In the current work, the ratio of fracture energy of FRP-confined specimens to that of unconfined specimens was taken as the ductility index. The area under the stress–strain curve gives the measure of the energy absorbed. Thus, an enhancement in ductility confirms the upsurge in confinement effect. In this manner, the suitable confinement of a structure enhances the ductility, augmenting energy dissipation capacity and thereby providing sufficient alerts before failure. The ductility and energy absorption values corresponding to unconfined and confined specimens when exposed to different temperature regimes are reported in Table 7. Evidently, the unconfined specimens recorded an energy absorption value of 4.416 MPa which is much below that of confined specimens. While MWCNT-modified HSBFRP specimens exhibit a fracture energy of 7.8, 7.82, 7.28, 6.97 and 6.74 times that of unconfined specimens when exposed to room conditions, 100 °C, 200 °C, 300 °C, and 400 °C respectively. The MWCNT-modified SFRP specimens exhibited a fracture energy of 5.43, 5.93, 5.58,5.3 and 5.04 times than that of unconfined specimens when exposed to different temperature regimes.

## 4. ANOVA

A GLM-ANOVA (general linear model analysis of variance) was employed at 0.05 significance level to evaluate the difference in the behavior of unconfined specimens, and confined specimens, when exposed to various elevated temperature regimes. In this manner, the type of FRP confinement was assigned as the dependent variable while the exposed room temperature, 100 °C, 200 °C, 300 °C, and 400 °C conditions were considered as the independent variable. A statistically significant element analysis was carried out at *p* level < 0.05.

When *p*-value is found to be less than 0.05, this indicates that the assumed variable is a significant factor in the study. Table 8 contains the results of statistical confirmation for selecting models using ANOVA. When the compressive strength of specimens exposed to room temperature and those exposed to 100 °C are compared, it is clear that the difference is not significant. Similarly, the compressive strength of specimens exposed to room temperature, 200 °C, 300 °C and 400 °C were compared, and it was concluded that a considerable variation exists. If the observed value is greater than 0.05, this indicates that the effect of that particular parameter is negligible when the performance of specimens is considered.

**Figure 13:**
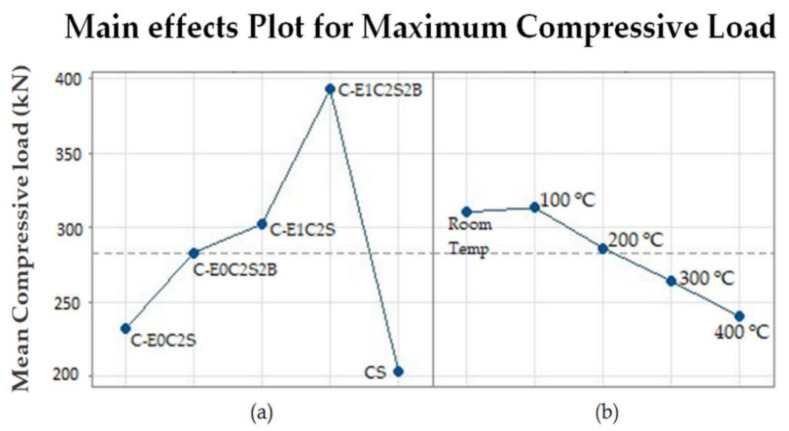
Main effects plot for maximum compressive load of specimens (**a**) Type of confinement (**b**) Exposure temperature.

A main effect plot was derived to analyze the individual influence of MWCNT modification, type of confinement and exposure temperature on the performance of specimens as seen in Figure 13. It may be noted that with respect to the unconfined specimens, the mean compressive strength exhibited by all the confined specimens other than C-E0C2S exceeded the mean value (denoted as dotted line) as evidence of the significant influence of FRP confinement on the enhanced axial compressive strength. The most significant and influencing factor observed was the type of FRP confinement when exposed to different elevated temperature conditions. The specimens confined with MWCNT-incorporated epoxy were found to have a superior performance when compared with neat epoxy-confined specimens. On the other hand, specimens confined with SFRP and HSBFRP had a greater influence on resistance to elevated temperatures until 200 °C, as inferred from the main effect plot curve. Such confinements can be used as rehabilitation materials subjected to elevated temperature conditions.

## 5. Conclusions

From the systematic analysis of the performance of unconfined and confined concrete specimens exposed to elevated temperature, the following conclusions were drawn:

- The bonding and strength of the epoxy-based FRP materials was found to be improved by the incorporation of MWCNT. An enhancement in tensile strength by 50% and 170%, respectively, was observed for E1C and E1C2S2B with the addition of MWCNT and fibers as fillers;
- All SFRP and HSBFRP-confined specimens exhibited enhanced strength when compared to unconfined specimens. The increment was found to be between 30 to 70% and 60 to 100% for SFRP and HSBFRP-confined specimens, respectively. This proves the effectiveness of hybrid fiber system (HSBFRP) as an external confinement;
- The unconfined cylinders exhibited a brittle failure upon exposure to elevated temperature whereas HSBFRP-confined specimens manifested a ductile failure due to the presence of FRP layers. In HSBFRP-confined specimens, the outer basalt FRP layer protects the internal sisal layers;
- The SFRP-confined specimens are adversely affected at elevated temperatures as evidenced from the color change caused due to the melting of FRP layers with a considerable reduction in compressive strength by 44% at 400 °C. On the other hand, the strength of MWCNT-incorporated HSBFRP-confined specimens at 400 °C reduced only by 22% in comparison with those at room temperature.

Overall, it may be concluded that, when exposed to elevated temperature conditions, the axial load carrying members made of concrete manifest a sudden reduction in strength. In such situations, concrete members confined with MWCNT-modified epoxy-based hybrid fiber systems can be employed for structural integrity and retention of compressive strength. The use of natural fibers, such as sisal and basalt, provides an alternative solution to the large-scale consumption of artificial fibers in the construction sector for strengthening/retrofitting applications.

## Figures and Tables

**Table 1:** Concrete mix ratio.

Constituents	Cement	FA	CA	W/C
Quantity	438.1	655.72	1130.29	197.16
Mix Ratio	1	1.50	2.58	0.45

**Table 2:** Summary of test specimens and temperature regimes.

	Type of Confinement	Type of Confinement	Type of FRP	Specimen Nomenclature	No of Specimens	Exposure Time (h)
Room temperature	Unconfined specimens	Unconfined specimens	-	CS	3	2
	Epoxy based confined specimens	Epoxy based confined specimens	SFRP	C-E0C2S	3	2
			HSBFRP	C-E0C2S2B	3	2
	Epoxy filled with MWCNT based confined specimens	MWCNT-incorporated epoxy-based confined specimens	SFRP	C-E1C2S	3	2
			HSBFRP	C-E1C2S2B	3	2
Temperature Exposure = 100 °C	Unconfined specimens	Unconfined specimens	-	CS100	3	2
	Epoxy based confined specimens	Epoxy based confined specimens	SFRP	C-E0C2S100	3	2
			HSBFRP	C-E0C2S2B100	3	2
	Epoxy filled with MWCNT based confined specimens	MWCNT-incorporated epoxy-based confined specimens	SFRP	C-E1C2S100	3	2
			HSBFRP	C-E1C2S2B100	3	2
Temperature Exposure = 200 °C	Unconfined specimens	Unconfined specimens	-	CS200	3	2
	Epoxy based confined specimens	Epoxy based confined specimens	SFRP	C-E0C2S200	3	2
			HSBFRP	C-E0C2S2B200	3	2
	Epoxy filled with MWCNT based confined specimens	MWCNT-incorporated epoxy-based confined specimens	SFRP	C-E1C2S200	3	2
			HSBFRP	C-E1C2S2B200	3	2
Temperature Exposure = 300 °C	Unconfined specimens	Unconfined specimens	-	CS300	3	2
	Epoxy based confined specimens	Epoxy based confined specimens	SFRP	C-E0C2S300	3	2
			HSBFRP	C-E0C2S2B300	3	2
	Epoxy filled with MWCNT based confined specimens	MWCNT-incorporated epoxy-based confined specimens	SFRP	C-E1C2S300	3	2
			HSBFRP	C-E1C2S2B300	3	2
Temperature Exposure = 400 °C	Unconfined specimens	Unconfined specimens	-	CS400	3	2
	Epoxy based confined specimens	Epoxy based confined specimens	SFRP	C-E0C2S400	3	2
			HSBFRP	C-E0C2S2B400	3	2
	Epoxy filled with MWCNT based confined specimens	MWCNT-incorporated epoxy-based confined specimens	SFRP	C-E1C2S400	3	2
			HSBFRP	C-E1C2S2B400	3	2

**Table 3:** Tensile properties of epoxy and multiscale composites.

Specimen	Tensile Strength (MPa)
E	40.2 ± 1.2
E1C	64.3 ± 1.8
E0C2S	72.9 ± 2.1
E1C2S	87.5 ± 2.4
E0C2S2B	76.3 ± 2.5
E1C2S2B	102.3 ± 3.2

**Table 4:** Compression test results of unconfined specimens exposed to elevated temperature.

Sl No	Specimen	Axial Compressive Strength (MPa) (f’_co_)
1	CS	14.3 ± 0.4
2	CS100	14.9 ± 0.2
3	CS200	13.6 ± 0.4
4	CS300	12.3 ± 0.5
5	CS400	11.0 ± 0.3

**Table 5:** Compression test results of SFRP-confined specimens exposed to elevated temperature.

Sl No	Confinement	Axial Compressive Strength (MPa) (f’_cc_ or f’_co_)	Confinement Effectiveness f’_cc_/f’_co_
1	C-E0C2S	16.9 ± 0.5	1.18
2	C-E1C2S	20.8 ± 0.6	1.45
3	C-E0C2S100	16.2 ± 0.2	1.09
4	C-E1C2S100	21.4 ± 0.4	1.43
5	C-E0C2S200	14.9 ± 0.5	1.10
6	C-E1C2S200	19.5 ± 0.3	1.43
7	C-E0C2S300	14.3 ± 0.6	1.16
8	C-E1C2S300	17.8 ± 0.4	1.53
9	C-E0C2S400	12.9 ± 0.5	1.18
10	C-E1C2S400	14.5 ± 0.6	1.59

**Table 6:** Compression test results of HSBFRP-confined exposed to elevated temperature.

Sl No	Confinement	Axial Compressive Strength (MPa) (f’_cc_ or f’_co_)	Confinement Effectiveness f’_cc_/f’_co_
1	C-E0C2S2B	21.4 ± 0.5	1.50
2	C-E1C2S2B	26.3 ± 0.6	1.91
3	C-E0C2S2B100	21.1 ± 0.3	1.71
4	C-E1C2S2B100	27.9 ± 0.5	1.87
5	C-E0C2S2B200	18.2 ± 0.3	1.33
6	C-E1C2S2B200	26.6 ± 0.6	1.95
7	C-E0C2S2B300	16.2 ± 0.4	1.32
8	C-E1C2S2B300	24.0 ± 0.5	1.95
9	C-E0C2S2B400	14.9 ± 0.3	1.35
10	C-E1C2S2B400	21.4 ± 0.4	1.96

**Table 7:** Energy ductility index of confined and unconfined specimens subjected to elevated temperatures.

Sl No	Specimen	Axial Compressive Strain (%)	Energy Absorbed (MPa)	Energy Ductility Index
1	CS	0.59 ± 0.06	4.4 ± 0.3	1.00 ± 0.06
2	C-E0C2S	1.06 ± 0.05	12.7 ± 0.4	2.87 ± 0.10
3	C-E0C2S2B	1.41 ± 0.09	22.4 ± 0.6	5.07 ± 0.20
4	C-E1C2S	1.50 ± 0.10	23.9 ± 0.5	5.43 ± 0.25
6	C-E1C2S2B	1.63 ± 0.11	34.4 ± 0.9	7.80 ± 0.30
7	CS100	0.59 ± 0.05	4.7 ± 0.2	1.00 ± 0.05
8	C-E0C2S100	1.07 ± 0.20	12.7 ± 0.30	2.69 ± 0.35
10	C-E0C2S2B100	1.48 ± 0.40	23.0 ± 1.20	4.87 ± 0.40
11	C-E1C2S100	1.54 ± 0.30	26.2 ± 1.80	5.54 ± 0.55
12	C-E1C2S2B100	1.70 ± 0.50	34.5 ± 2.30	7.30 ± 0.60
14	CS200	0.34 ± 0.05	4.0 ± 0.15	1.00 ± 0.05
15	C-E0C2S200	1.12 ± 0.20	17.3 ± 0.90	4.28 ± 0.15
16	C-E0C2S2B200	1.52 ± 0.30	21.4 ± 1.20	5.32 ± 0.30
17	C-E1C2S200	1.58 ± 0.25	24.6 ± 1.45	6.11 ± 0.45
18	C-E1C2S2B200	1.74 ± 0.35	32.1 ± 1.50	7.97 ± 0.35
19	CS300	0.32 ± 0.04	3.7 ± 0.15	1.00 ± 0.05
20	C-E0C2S300	1.16 ± 0.06	12.1 ± 0.60	3.23 ± 0.09
21	C-E0C2S2B300	1.54 ± 0.09	20.3 ± 0.85	5.40 ± 0.20
22	C-E1C2S300	1.61 ± 0.15	23.4 ± 0.95	6.23 ±0.25
23	C-E1C2S2B300	1.78 ± 0.20	30.8 ± 0.90	8.19 ± 0.45
24	CS400	0.31 ± 0.03	3.1 ± 0.10	1.00 ± 0.02
25	C-E0C2S400	1.17 ± 0.25	11.2 ± 0.95	3.61 ± 0.09
26	C-E0C2S2B400	1.57 ± 0.30	18.2 ± 1.20	5.85 ± 0.15
27	C-E1C2S400	1.63 ± 0.35	22.2 ± 1.50	7.17 ± 0.25
28	C-E1C2S2B400	1.85 ± 0.40	29.3 ± 1.60	9.45 ± 0.35

**Table 8:** Analysis of variance for exposure temperature.

Exposure Temperature	Adj Sum of Squares	Adj Mean Square	F-Value	*p*-Value	Significance
100 °C	22.5	22.5	0.47	0.52	No
200 °C	1440	1440	10.29	0.03	Yes
300 °C	5290	5290	24.60	0.00	Yes
400 °C	12,250	12,250	44.55	0.00	Yes

## Data Availability

Not applicable.
